# RIPK3 activation promotes DAXX‐dependent neuronal necroptosis after intracerebral hemorrhage in mice

**DOI:** 10.1111/cns.14397

**Published:** 2023-08-08

**Authors:** Qingqing Bai, Shuoyang Wang, Dongmei Rao, Zhiming Zhou, Jianfei Wang, Qi Wang, Yu Qin, Zhaohu Chu, Shoucai Zhao, Dijing Yu, Yang Xu

**Affiliations:** ^1^ Department of Neurology First Affiliated Hospital of Wannan Medical College, Yijishan Hospital Wuhu Anhui China; ^2^ Anhui Province Key Laboratory of Non‐coding RNA Basic and Clinical Transformation Wannan Medical College Wuhu Anhui China; ^3^ Department of Ophthalmology Wuhu Eye Hospital Wuhu Anhui China

**Keywords:** death domain‐associated protein, intracerebral hemorrhage, necroptosis, neurobehavioral deficits, receptor‐interacting protein kinase 3

## Abstract

**Background:**

Necroptosis induced by receptor‐interacting protein kinase 3 (RIPK3) is engaged in intracerebral hemorrhage (ICH) pathology. In this study, we explored the impact of RIPK3 activation on neuronal necroptosis and the mechanism of the death domain‐associated protein (DAXX)‐mediated nuclear necroptosis pathway after ICH.

**Methods:**

Potential molecules linked to the progression of ICH were discovered using RNA sequencing. The level of DAXX was assessed by quantitative real‐time PCR, ELISA, and western blotting. DAXX localization was determined by immunofluorescence and immunoprecipitation assays. The RIPK3 inhibitor GSK872 and DAXX knockdown with shRNA‐DAXX were used to examine the nuclear necroptosis pathway associated with ICH. Neurobehavioral deficit assessments were performed.

**Results:**

DAXX was increased in patients and mice after ICH. In an ICH mouse model, shRNA‐DAXX reduced brain water content and alleviated neurologic impairments. GSK872 administration reduced the expression of DAXX. shRNA‐DAXX inhibited the expression of p‐MLKL. Immunofluorescence and immunoprecipitation assays showed that RIPK3 and AIF translocated into the nucleus and then bound with nuclear DAXX.

**Conclusions:**

RIPK3 revitalization promoted neuronal necroptosis in ICH mice, partially through the DAXX signaling pathway. RIPK3 and AIF interacted with nuclear DAXX to aggravate ICH injury.

## Graphical abstract

RIPK3 induces AIF expression and nucleus translocation after ICH. Then, RIPK3 and AIF were combined with DAXX, respectively. DAXX‐RIPK3 and DAXX‐AIF complexes contribute to MLKL phosphorylation. RIPK3 inhibitor GSK872 suppresses the necroptosis cascade and attenuates AIF, DAXX, and MLKL expression. When RIPK3 is inhibited, DAXX‐RIPK3 and DAXX‐AIF binding disappeared.

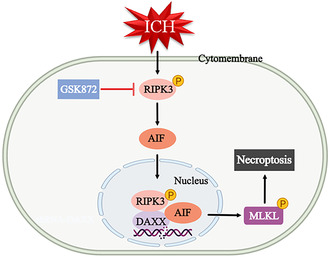

## 1 INTRODUCTION

Intracerebral hemorrhage (ICH) is a cerebral vascular sickness that causes severe morbidity and mortality, accounting for 15% of strokes worldwide.^1^, ^2^ The hematoma mass effect and mechanical injury cause neurologic impairments associated with ICH.^3^ Secondary brain injury inhibits the functions of nerves by causing brain swelling, which results in the expulsion of harmful substances in the blood and inflammatory reactions that induce cell death in the brain and brain damage after ICH.^4^ As a result, strategies for avoiding cell death might be helpful in the treatment of ICH.

Necroptosis is a kind of programmed necrotic cell death induced by death receptors (DRs).^5^ RIPK1 and RIPK3 are phosphorylated, which activates their kinase effects.^6^ The mixed lineage kinase domain‐like protein (MLKL) is phosphorylated, which causes MLKL to move and polymerize in cell membranes, ultimately leading to the expansion of cells and the breakdown of membranes.^7^ In addition to RIPK1 and RIPK3, apoptosis‐inducing factor (AIF) regulates necroptosis.^8^ Depolarized mitochondria release AIF, which then moves to the nucleus. It is associated with chromatin condensation and chromatolysis.^9^ Yuan et al. discovered that RIPK3 levels were elevated in the brains of ICH mice, and MLKL phosphorylation and oligomerization were observed, demonstrating that necroptosis occurs in ICH brains.^10^ However, there has not been more research done to date on the primary cause and associated mechanism of necroptosis activation in ICH brains.

According to some research, death‐associated protein (DAXX) is a preserved nuclear protein. DAXX is expressed in neurons of the brain.^11^ Rat hippocampal CA1 neurons were protected from cerebral ischemia/reperfusion after DAXX trafficking was blocked.^12^ Recent research has also shown that DAXX inhibition prevents neuronal cell death and ischemic brain injury in mice after focal cerebral ischemia.^13^ According to publications, the DAXX protein is a member of the proteins in the RIPK3 signaling downward pathway. Neuronal RIPK3 suppression prevents DAXX transport from the nucleus to the cytosol. It causes DAXX suppression during ischemia/reperfusion damage in rats.^14^ RIPK3 phosphorylates DAXX at Ser‐668 in ganglion cells of the retina during ischemic cell death, causing DAXX nuclear export.^15^ Although DAXX promotes brain injury responses, its effects on RIPK3‐mediated neuronal necroptosis in the nucleus have not been examined after ICH. In this study, we proposed that RIPK3 stimulation activated the nuclear DAXX‐mediated necroptosis pathway after ICH.

## 2 METHODS

### 2.1 Ethics, participants and animals

Human samples were collected following protocols allowed by the Wannan Medical College's Medical Ethics Committee (2020–30). The Wannan Medical College Ethics Committee on Experimental Animals approved all methods of animal experimentation utilized in this study (LLSC2020040). Patients with ICH were recruited from Yijishan Hospital's Department of Neurology, Wannan Medical College. Individuals receiving a wellness examination were recruited as healthy control volunteers. A written consent form was signed by participants or their legally appointed representatives. Additional details are provided in the Supplementary Information.

All adult male C57BL/6 mice weighed 25–30 g, were 8–10 weeks old, and were purchased from Nanjing Qinglong Mountain Farm for Animals. The animals stayed in a quiet space (interior temperature range of 18–22°C) and fed freely. The studies were conducted in a blinded manner, and all of the mice underwent surgery carried out by the same surgeon.

### 2.2 ICH Model Induction

A previously reported method was used to build the ICH model.^16^ Isoflurane was used to anesthetize mice. At a depth of 3.0 mm, collagenase IV was administered (0.075 U in 500 nL of saline). A hole was cut in the skull that measured 0.5 mm in diameter, 0.2 mm from the bregma, and 2.0 mm from the middle to the right. The sham group received the same operation but with an equivalent volume of saline instead of collagenase. Mice were kept at a temperature of 37 ± 0.5°C during the procedure and recuperation period. 4% isoflurane was used for anesthetic induction, while 2% isoflurane was administered for brain operations. The flow of oxygen was 3 L/min.

### 2.3 Experimental protocol

All experimental protocols were provided in the Supplementary Information. The four distinct experiments shown in the experimental design timeline in Figure S1 were allotted to the mice at random.

### 2.4 Intraventricular injection and drug administration

A 25 mM solution of GSK872 (HY‐101872, MedChemExpress) was dispersed in 1% dimethyl sulfoxide (DMSO). Each mouse's homolateral cerebral ventricle received 4 μL of ready GSK872 at 1 h before ICH. shRNA‐DAXX (GenePharma, 1 × 10^9^ TU/ml) was administered via intracerebroventricular injection 2 weeks before ICH (1 μL per mouse). Over the course of 10 min, the drug was progressively injected using a microliter syringe at the locations (2.7 mm deep, 1.0 mm anterior to the bregma). Control mice were given an equal volume of vehicle.

### 2.5 Assessment of neurological deficits

#### 2.5.1 Garcia test

The test is divided into seven portions, comprising the assessment of forelimb walking, climbing, spontaneous motion, axis feeling, vibrissae proprioception, mobility of limb symmetry, and lateral rotation.

#### 2.5.2 Corner turn testing

The mice were positioned at a 30° angle. To exit, a mouse will turn to the left or the right. The percentages of left turns were noted.

#### 2.5.3 Morris water maze

The water maze projects were completed as previously mentioned.^17^ Over the course of 5 days, the mice were trained daily to locate a submerged white platform and carried out four experiments. After the training tests were finished on day 5, the platform was taken away to inspect the probe, and the mice were made to swim to the previous platform's position. The entire set of data was analyzed using the Smart 3.0 program (Panlab).

### 2.6 Brain edema

The brain was separated into three sections: cerebellum, left brain, and right brain. To calculate the wet weight, each component was weighed. After being weighed, the tissue sections were left to dry for 24 h at 100°C: (wet weight ‐ dry weight)/wet weight × 100% equals brain water content.

### 2.7 Quantitative real‐time PCR

Total RNA was obtained and quantified using spectrophotometry from mouse right brain perihematomal tissues and serum of ICH patients using TRIzol. A Prime Script RT reagent kit and an SYBR Green kit (both supplied by Tian Gen) were put to use for reversible transcription and subsequent real‐time PCR analysis. Ribo Biotechnology provided the following mouse DAXX and GAPDH primers: DAXX, forward 5’‐ACCCAGACTCCTCGTAT‐TTGC‐3′, reverse 5’‐TTCGCTGCTCTATG‐ACCCG‐3′; GAPDH, forward 5’‐GAACGGGAAGCTCA‐CTGG‐3′, reverse 5’‐GCCTGCTTCACCACCTT‐CT‐3′. Sangon Biotechnology produced the human primers DAXX and GAPDH mentioned below: DAXX, forward 5’‐ACCGCTAA‐CAGCATCATCGT‐3′, reverse 5’‐ATTTCTTGCCGCCCGAACTA‐3′; RIPK3, forward 5'‐ATCTAGAGGAGCCTCCCAGC‐3', RIPK3, reverse 5'‐GGTTGGGCCATCGAATCTGA‐3'; GAPDH, forward 5’‐GAGAAGGCTGGGGCTCATTT‐3′, reverse 5’‐AGTGATGGCATGGA‐CTGTGG‐3′.

### 2.8 Western blot analysis

Right brain perihematomal tissues were collected from the mice 24 h after ICH. The WB analysis of the brain tissues was conducted as earlier mentioned.^8^ The membranes were then treated with rabbit antibody to RIPK3 (1:1000, A5431, ABclonal), rabbit antibody to DAXX (1:1000, A1642, ABclonal), rabbit antibody to AIF (1:1000, 17,984‐1‐AP, Proteintech), rabbit antibody to MLKL (1:1000, #14993, Cell Signaling Technology), rabbit antibody to p‐MLKL (1:1000, #37333, Cell Signaling Technology), rabbit antibody to actin (1:1000, Servicebio, GB11001), and rabbit antibody to H3 (1:1000, GB11102, Servicebio) through the night at 4°C. With ImageJ software, densitometric analysis was utilized to measure the relative intensity of each protein signal.

### 2.9 Enzyme‐linked immunosorbent assay

The human DAXX ELISA kit (SP39175, Saipei Biotechnology) was used to assess the concentration of DAXX in the serum of ICH patients. All measures were taken according to the manufacturer's protocols.

### 2.10 Immunoprecipitation

Antibodies (1–2 g) were administered to each nuclear protein from the right cerebral hemispheres of mice for 4 h at 4°C. Each sample was mixed with protein A/G agarose through the night at 4°C. After being cleaned and spit using SDS‐PAGE, immunoprecipitates were then blotted via the relevant antibodies.

### 2.11 Immunofluorescence

The staining of brain tissue sections from mice was carried out exactly as mentioned earlier.^18^ The following primary antibodies were mixed with cerebral slices at 4°C for an entire night: mouse antibody to NeuN (1:200, 66,836‐1‐Ig, Proteintech), rabbit antibody to RIPK3 (1:100, 17,563‐1‐AP, Proteintech), rabbit antibody to DAXX (1:100, AF5421, Affinity), rabbit antibody to AIF (1:100, 17,984‐1‐AP, Proteintech), and mouse antibody to DAXX (1:100, sc‐8043, Santa Cruz). The slides were immersed in Cy3‐labeled goat anti‐rabbit IgG (1:100, GB21303, Servicebio) or FITC‐labeled goat anti‐mouse IgG (1:100, GB22301, Servicebio) after being washed in PBS for 2 h under ambient conditions in the dark. DAPI (P0131, Beyotime) was used to stain the tissues. Slides were viewed and captured utilizing a fluorescence microscope (LSM880, ZEISS).

### 2.12 Quantitative RNA sequencing

Human peripheral blood was collected into EDTA tubes at 24 h after ICH. RNA‐Seq experiments were performed by LC Biotech. TRIzol reagent was employed to extract total RNA from whole blood. Reverse transcription was used to create cDNAs from the measured and extracted total RNA. These cDNAs served to synthesize U‐labeled second‐stranded DNAs. PCR was applied to amplify the ligated products, and the produced cDNA library had a mean insertion length of 300 bp (50 bp). The parameters for statistical levels were |log2 (Fold change) | ≥ 1 and *p* < 0.05.

### 2.13 Statistical analysis

The mean ± SEM for all data is displayed. GraphPad Prism version 7.0 was applied for the statistical analysis. All data were checked for Gaussian distribution using the Shapiro–Wilk test and then were analyzed by *t*‐test, one‐way ANOVA, or two‐way ANOVA depending on the circumstances. Data that did not have a normal/Gaussian distribution were analyzed via a nonparametric equivalent, and utilized by the post hoc test for pairwise comparison of the data. In statistics, *p* < 0.05 was regarded as meaningful.

## 3 RESULTS

### 3.1 DAXX expression was elevated in ICH patients

**FIGURE 1:**
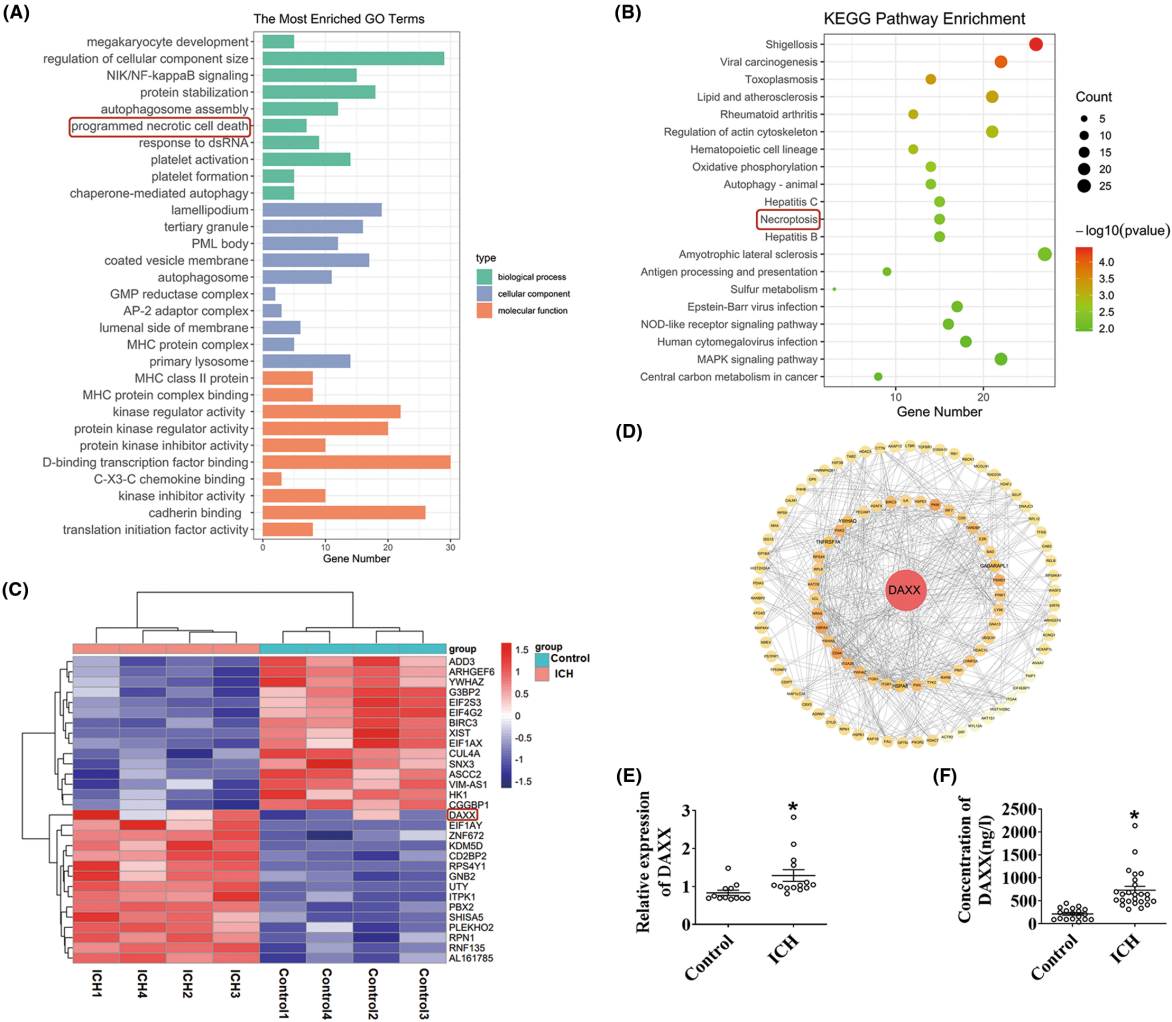
DAXX expression was elevated in ICH patients. (A) Gene Ontology enrichment analysis of differentially expressed genes in ICH patients (*n* = 4) and healthy controls (*n* = 4). The results are summarized in three main categories: biological process, molecular function, and cellular component. (B) Kyoto Encyclopedia of Genes and Genomes analysis of the differentially expressed ICH patients (*n* = 4) and healthy controls (*n* = 4). The top 20 KEGG clusters are shown with their keywords. (C) Heatmap showing differentially expressed genes in ICH patients (*n* = 4) and healthy controls (*n* = 4), and data are colored according to the row minimum and maximum. (D) Protein–protein interaction network analysis using the STRING database to evaluate DAXX in ICH patients (*n* = 4) and healthy controls (*n* = 4). (E) Quantitative real‐time PCR of DAXX mRNA expression in the serum of patients with ICH (*n* = 14) compared with that in healthy control participants (*n* = 12). GAPDH was used as an internal control. (F) Enzyme‐linked immunosorbent assay results showed the level of DAXX in the serum after ICH patients (*n* = 25) compared with that in healthy control participants (*n* = 18). *p < 0.05 compared with control.

We first used RNA‐seq in peripheral blood samples from ICH patients to identify the participants in the progression of this disease. Significant differences were determined by |log2 (Fold change) | ≥ 1 and *p* < 0.05. In total, 710 differentially expressed genes (DEGs) were found. Biological processes, cellular components, and molecular functions were all included in the Gene Ontology enrichment analysis. In particular, we noticed that programmed necrotic cell death participated in pathophysiological responses following ICH injury. There was a significant portion of genes in the BP category (Figure 1A). Pathway analysis of these 710 DEGs demonstrated that the necroptosis signaling pathway was one of the most enriched gene ontology category (Figure 1B). We found that ICH induced the expression of the DAXX gene, which promotes cell death (Figure 1C). We conducted a protein–protein interaction (PPI) analysis on these DEGs to further analyze their possible interactions. We discovered that DAXX was in the functional PPI network's hub positions and was tightly associated with others (Figure 1D, Figure S7). We analyzed DAXX levels in the peripheral blood of healthy individuals and patients with ICH to validate the RNA‐seq data. Table S1 contained demographic and clinical information about the participants. qRT‐PCR (Figure 1E) and ELISA (Figure 1F) showed that the ICH group had higher‐serum DAXX levels than the control group at 24 h. DAXX (Figure S2A,B) and RIPK3 (Figure S2C,D) were increased in the serum of ICH patients for a specific time. The box plot presents the distribution of DEG expression levels by fragments per kilobase of transcript sequence per millions of mapped reads (FPKM) in healthy controls and ICH patients (Figure S3A). The principal component analysis plot showed that the gene profiles of the healthy control and ICH groups were different (Figure S3B). These results ensured the reliability of the bioinformatics analysis.

### 3.2 ICH promoted endogenous levels of RIPK3, AIF, DAXX, MLKL and p‐MLKL in mice

**FIGURE 2:**
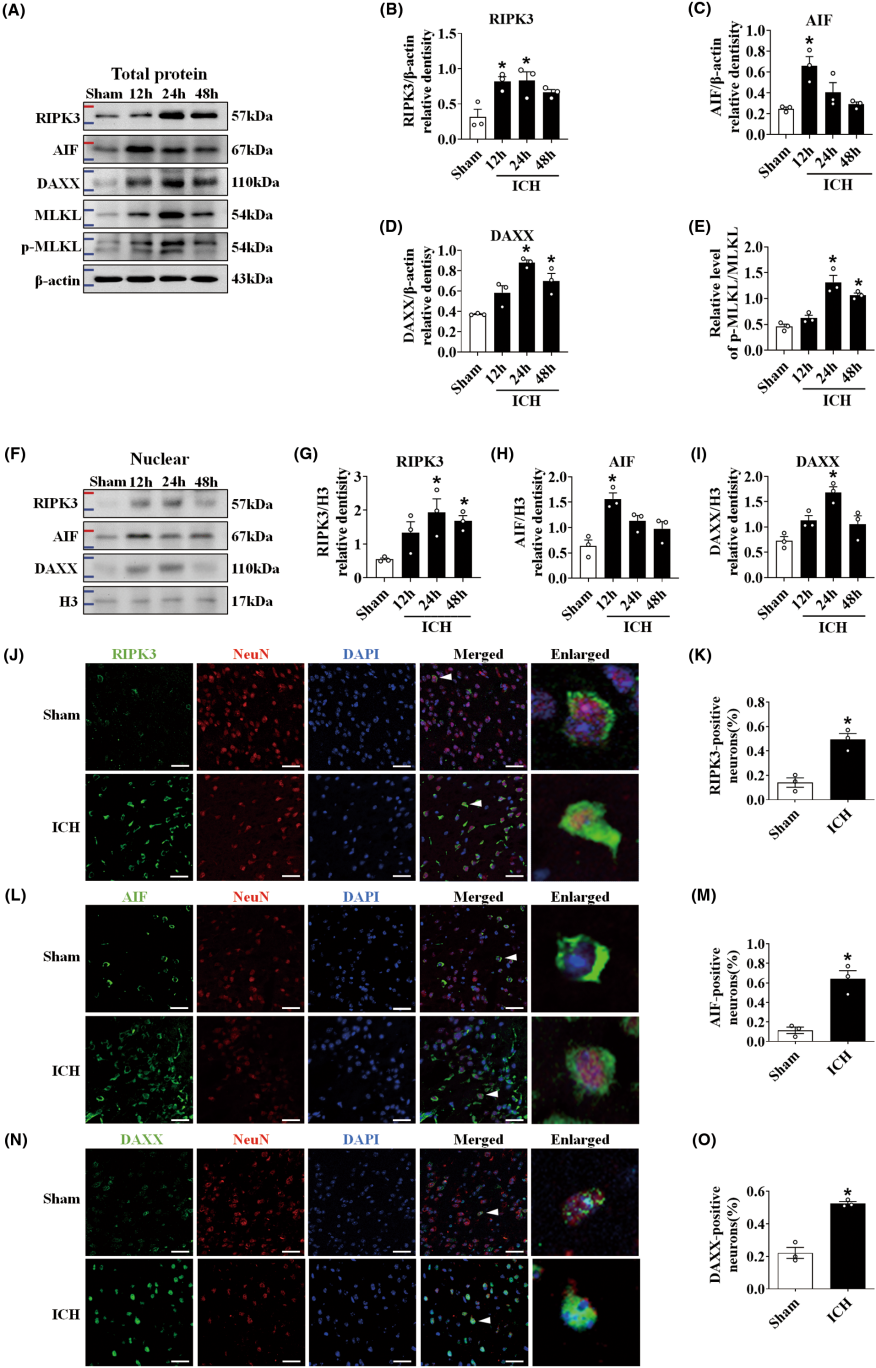
ICH promoted endogenous levels of RIPK3, AIF, DAXX, MLKL, and p‐MLKL in mice. (A) Representative western blot images. (B–D) Quantitative analyses of RIPK3, AIF, and DAXX at 12 h, 24 h, and 48 h in total proteins in the sham and ICH groups (*n* = 3/group). (E) The relative level of p‐MLKL/MLKL at 12 h, 24 h, and 48 h in the sham and ICH groups (*n* = 3/group). (F) Representative western blot images. (G–I) Quantitative analyses of RIPK3, AIF, and DAXX, at 12 h, 24 h, and 48 h in nuclear proteins in the sham and ICH groups (*n* = 3/group). (J–O) Representative immunofluorescent images and quantification of RIPK3 (green), AIF (green), and DAXX (green) with NeuN (red, neuron marker) at 24 h in the sham and ICH groups (*n* = 3/group). Scale bar = 50 μm. *p < 0.05 compared with sham.

We created time courses of 12 h, 24 h (Figure S4), and 48 h to investigate alterations in RIPK3, AIF, DAXX, MLKL, and p‐MLKL after ICH in mice. The ICH group showed higher levels of total RIPK3, AIF, DAXX, and p‐MLKL/MLKL expression than the sham group. RIPK3 (Figure 2B), DAXX (Figure 2D), and p‐MLKL/MLKL (Figure 2E) expression reached a high point at 24 h and declined at 48 h, but AIF (Figure 2C) expression reached its highest at 12 h and reduced at 24 h after ICH. The nuclear locations of RIPK3, AIF, and DAXX were of greater significance than those of other organelles. Western blotting revealed that the ICH group had higher nuclear levels of RIPK3, AIF, and DAXX than the control group. Nuclear RIPK3 (Figure 2G) and DAXX (Figure 2I) levels reached a high point at 24 h during ICH, and AIF (Figure 2H) expression reached its peak at 12 h during ICH. As a result, for a deeper look at the mechanism of ICH, the time point of 24 h was chosen.

Double immunoprecipitation staining was performed to examine RIPK3‐, AIF‐, and DAXX‐positive neurons in the brains of ICH mice. The sham group that just several staining‐positive cells persisted, while positive neurons were discovered in the perihematomal region in the ICH group. This result indicated that RIPK3, AIF, and DAXX were activated following ICH damage. For the sham group, green‐labeled RIPK3 and AIF were observed in a punctate manner surrounding their nucleus, green‐labeled DAXX was discovered in the cell nuclei, and DAPI interacted with red‐labeled NeuN in neuronal nuclei. For the ICH group, RIPK3 (Figure 2J,K), AIF (Figure 2L,M), and DAXX (Figure 2N,O) were strongly colabeled with NeuN, and the combined image showed that these were mostly expressed on the neurons of the ipsilateral basal cortex.

### 3.3 DAXX expression was positively regulated by RIPK3 after ICH induction

**FIGURE 3:**
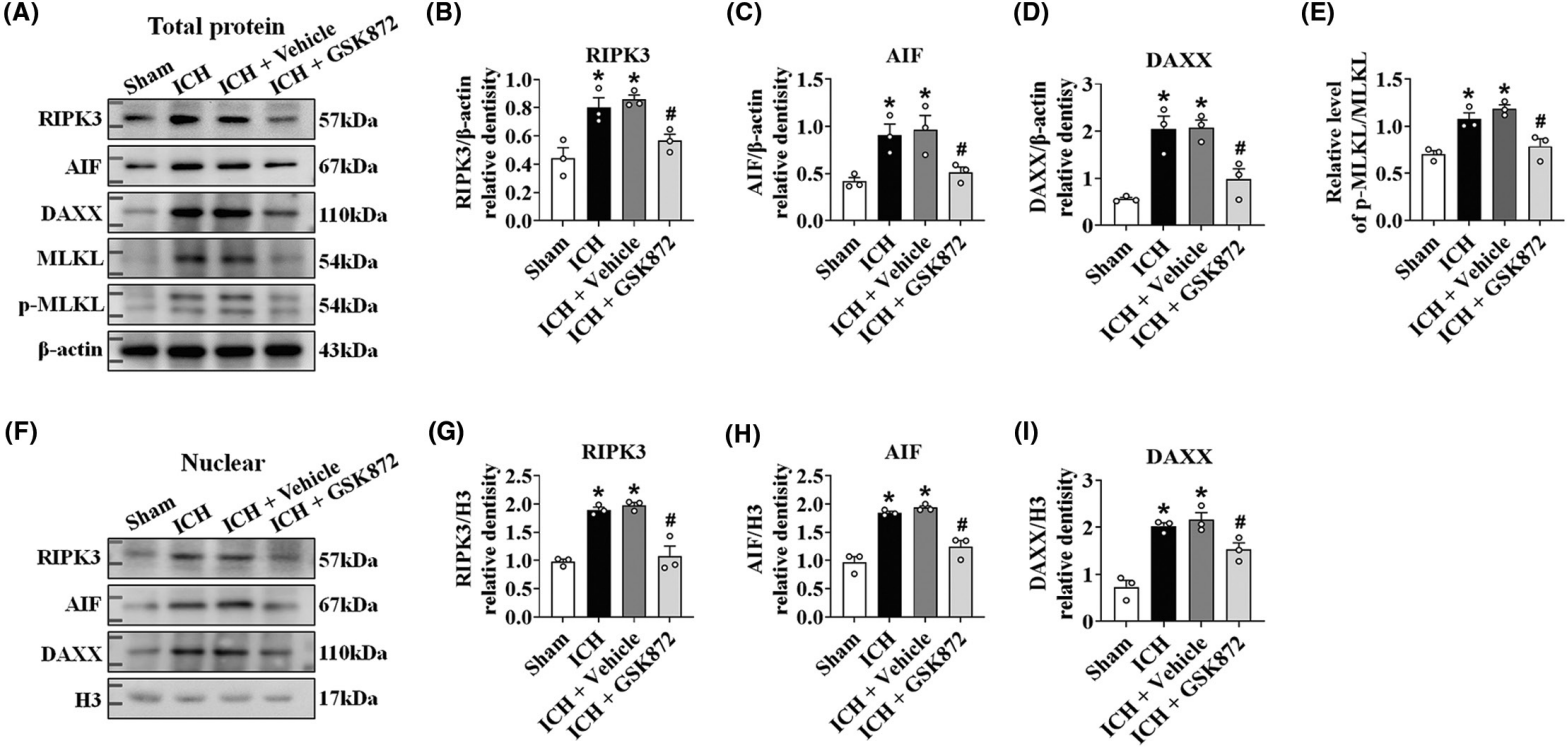
DAXX expression was positively regulated by RIPK3 after ICH induction. (A) Representative western blot bands. (B–D) Quantitative analyses of the total levels of RIPK3, AIF, and DAXX in the sham, ICH, ICH + vehicle, and ICH + GSK872 groups (*n* = 3/group). (E) The relative level of p‐MLKL/MLKL in the sham, ICH, ICH + vehicle, and ICH + GSK872 groups (*n* = 3/group). (F) Representative western blot bands. (G–I) Quantitative analyses of the nuclear levels of RIPK3, AIF, and DAXX after ICH in the sham, ICH, ICH + vehicle, and ICH + GSK872 groups (*n* = 3/group). *p < 0.05 compared with sham. ^#^ *p* < 0.05 compared with ICH + vehicle.

The RIPK3 inhibitor GSK872 was administered to ICH mice to confirm the downstream role of RIPK3. The GSK872 solution was prepared using 1% DMSO. This concentration of DMSO had no toxic effect on the brain (Figure S5). GSK872 administration dramatically reduced total and nuclear RIPK3 expression (Figure 3B,G). The ICH‐induced increases in total AIF (Figure 3C), DAXX (Figure 3D), and p‐MLKL/MLKL (Figure 3E) levels were reversed by inhibiting RIPK3. Pretreatment with GSK872 significantly reduced the expression of AIF (Figure 3H) and DAXX (Figure 3I) in the nucleus. These results suggested that the activated RIPK3 necroptosis pathway induced DAXX expression.

### 3.4 DAXX signaling promoted MLKL‐mediated necroptosis

**FIGURE 4:**
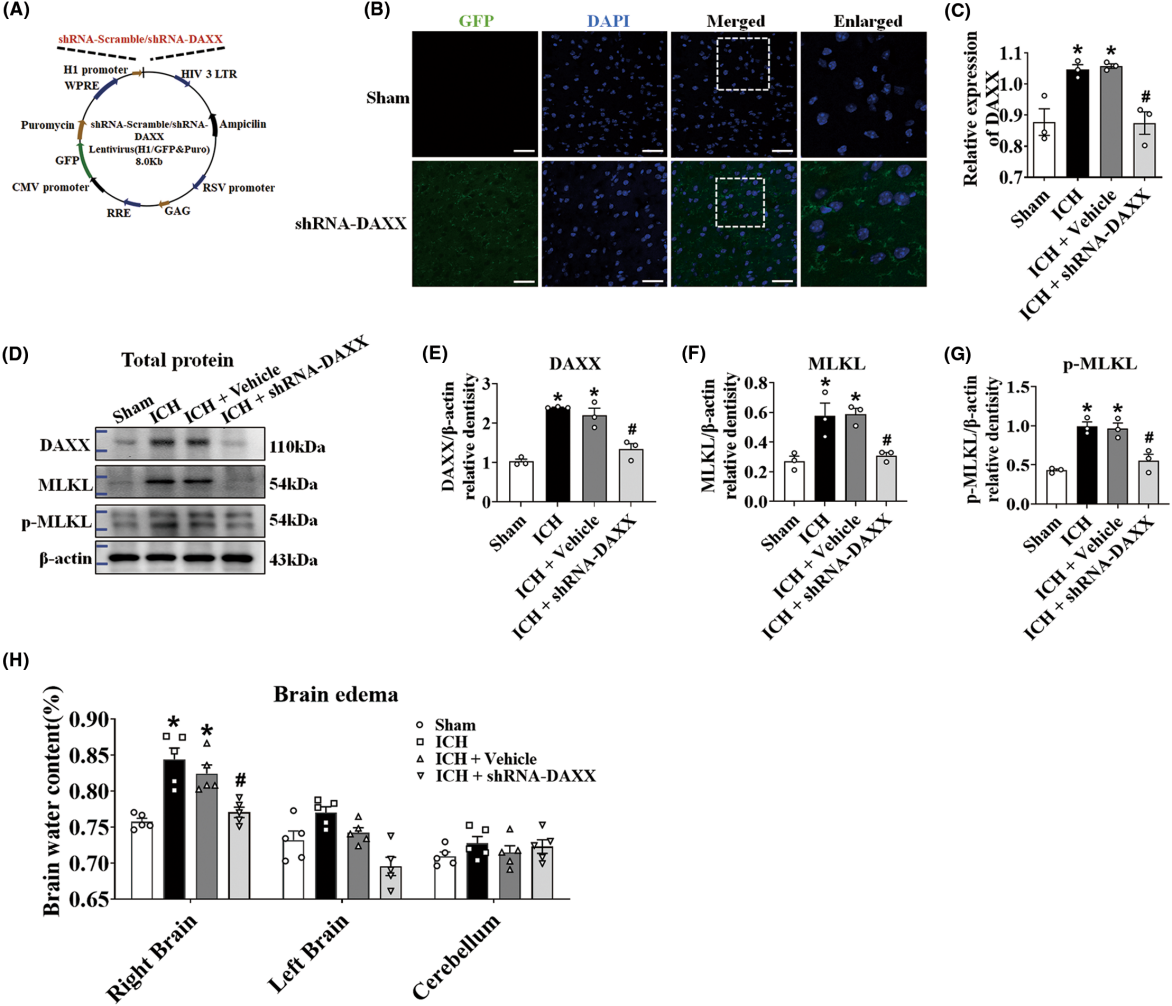
DAXX signaling promoted MLKL‐mediated necroptosis. (A) Schematic showing the lentiviral vector encoding DAXX. (B) Representative images of the mouse lateral ventricle microinjected with fluorescent (GFP) lentiviruses. Mice were killed 2 weeks after microinjection, and GFP expression was measured in the sham and shRNA‐DAXX groups (*n* = 3/group). Scale bar = 50 μm. (C) Quantitative real‐time PCR of DAXX mRNA expression in the sham, ICH, ICH + vehicle, and ICH + shRNA‐DAXX groups (*n* = 3/group). (D) Representative Western blot images. (E–G) Quantitative analyses of DAXX, MLKL, and p‐MLKL in the sham, ICH, ICH + vehicle, and ICH + shRNA‐DAXX groups (*n* = 3/group). (H) Brain edema at 24 h in the sham, ICH, ICH + vehicle, and ICH + shRNA‐DAXX groups (*n* = 5/group). *p < 0.05 compared with sham. ^#^ *p* < 0.05 compared with ICH + vehicle.

To investigate the direct contribution of DAXX signaling to the activation of MLKL‐mediated necroptosis, we examined the effect of DAXX suppression in ICH mice. The lateral ventricle was microinjected with either shRNA‐Scramble‐GFP or shRNA‐DAXX‐GFP lentiviruses (Figure 4A). The transduction efficiency was confirmed by fluorescence microscopy, WB, and qRT‐PCR in HT22 cells (Figure S6). The data showed that shRNA‐DAXX#1 treatment reduced DAXX expression in HT22 cells. We further validated these observations in the cellular context of a mouse. shRNA‐DAXX#1 was administered to the mice via intracerebroventricular injection for 2 weeks. In the shRNA‐DAXX group of mice, GFP lentivirus expression was largely observed throughout the entire brain (Figure 4B). In the ICH + shRNA‐DAXX group, DAXX expression was dramatically reduced in brain tissues (Figure 4C,E). In the ICH + shRNA‐DAXX group, MLKL and p‐MLKL levels fell below the ICH group (Figure 4F,G). There was an increase in the percentage of perihematomal brain water in the ICH and ICH + vehicle groups. shRNA‐DAXX treatment markedly reduced brain edema after ICH (Figure 4H). These results suggested that knockdown DAXX deregulated MLKL‐mediated necroptosis after ICH.

### 3.5 RIPK3 and AIF combined with DAXX respectively in the nucleus after ICH

**FIGURE 5:**
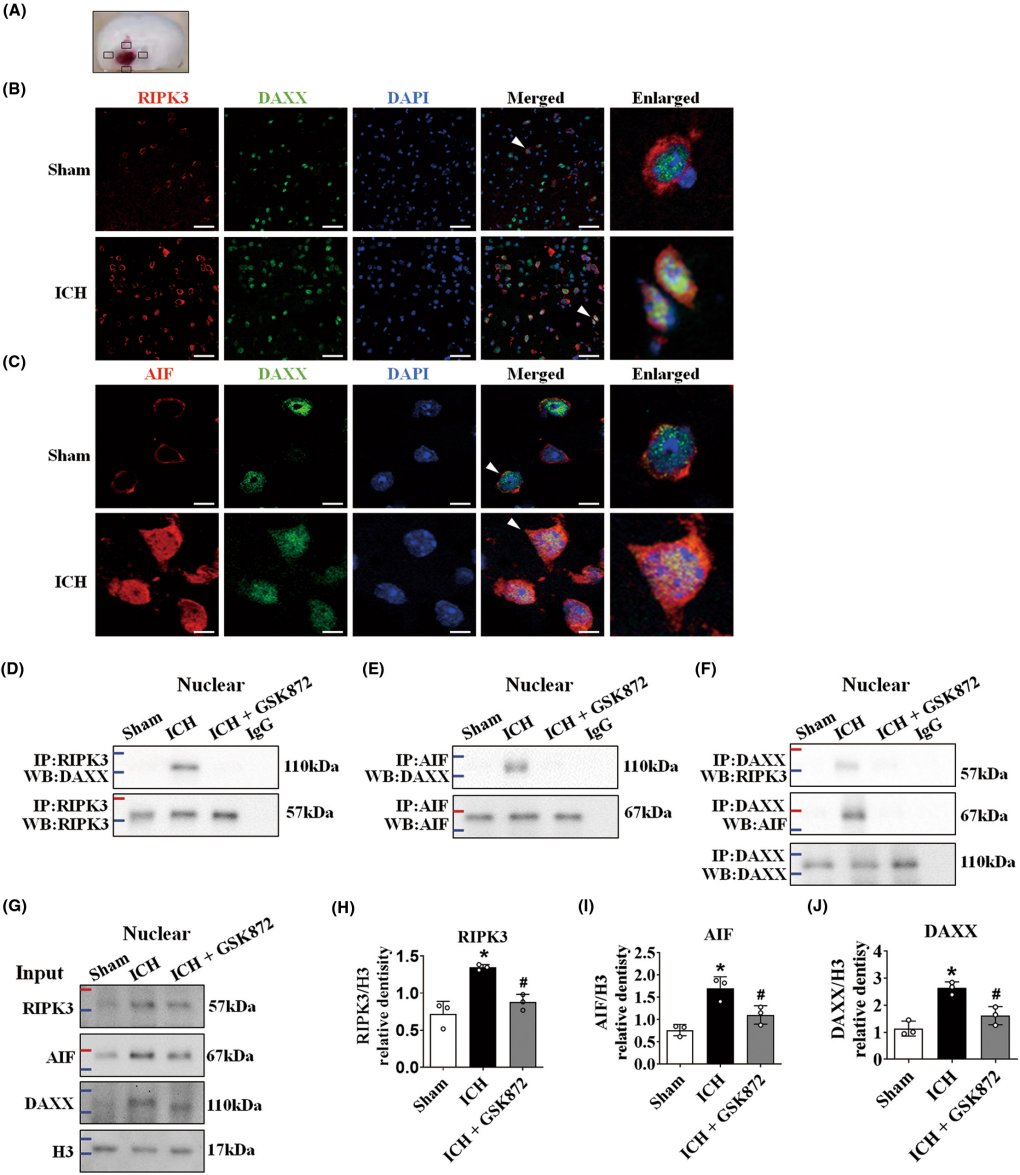
RIPK3 and AIF combined with DAXX, respectively, in the nucleus after ICH. (A) The small black square in the coronal section of the brain indicates the area where the microphotograph was taken. (B) Immunofluorescence staining showed the expression of DAXX (green) and RIPK3 (red) in the perihematomal area in the sham and ICH (24 h) groups (*n* = 3/group). Scale bar = 50 μm. (C) Immunofluorescence staining showed the expression of DAXX (green) and AIF (red) in the perihematomal area in the sham and ICH (24 h) groups (*n* = 3/group). Scale bar = 10 μm. (D–G) Representative immunoprecipitation results and input protein levels of RIPK3, AIF, and DAXX in the nuclear protein fraction after ICH injury. (H–J) Quantitative analyses of RIPK3, AIF, and DAXX in the nuclear protein fraction in the sham, ICH, and ICH + GSK872 groups (*n* = 3/group). *p < 0.05 compared with sham. ^#^ *p* < 0.05 compared with ICH.

Immunofluorescence staining was carried out in the brain tissue around the hematoma at 24 h post‐ICH (Figure 5A). The cytoplasm in the sham group showed RIPK3 and AIF fluorescence primarily surrounding the nucleus. DAXX fluorescence was spread throughout the nucleus. The DAPI‐stained nuclei were spherical, with moderately diffuse staining. Conversely, DAPI staining revealed a markedly compacted nuclear morphology in the ICH group. In addition to being intensified around the nucleus, RIPK3, and AIF fluorescence signals were dispersed inside the nucleus. The immunofluorescence intensity of RIPK3 and AIF staining were detected in pyknotic neuronal nuclei with concentrated DAPI staining after ICH. DAXX locations were scattered throughout the nucleus in both the sham group and the ICH group. Interestingly, DAXX colocalized with not only RIPK3 (Figure 5B) but also AIF (Figure 5C) in the nucleus. We isolated nuclear proteins and used immunoprecipitation to identify interacting proteins. In the sham group, no immunoprecipitation bands were observed. The ICH group showed nuclear DAXX, RIPK3, and AIF bands (Figure 5D–F), indicating the complex was assembled following ICH. In the ICH + GSK872 group, there were no nuclear protein bands (Figure 5D–F). To determine whether they worked, we utilized RIPK3 (Figure 5H), AIF (Figure 5I), and DAXX (Figure 5J) in nuclear fraction as input. These factors were expressed in the ICH group and inhibited by GSK872.

### 3.6 shRNA‐DAXX treatment alleviated the neurological impairments caused by ICH

**FIGURE 6:**
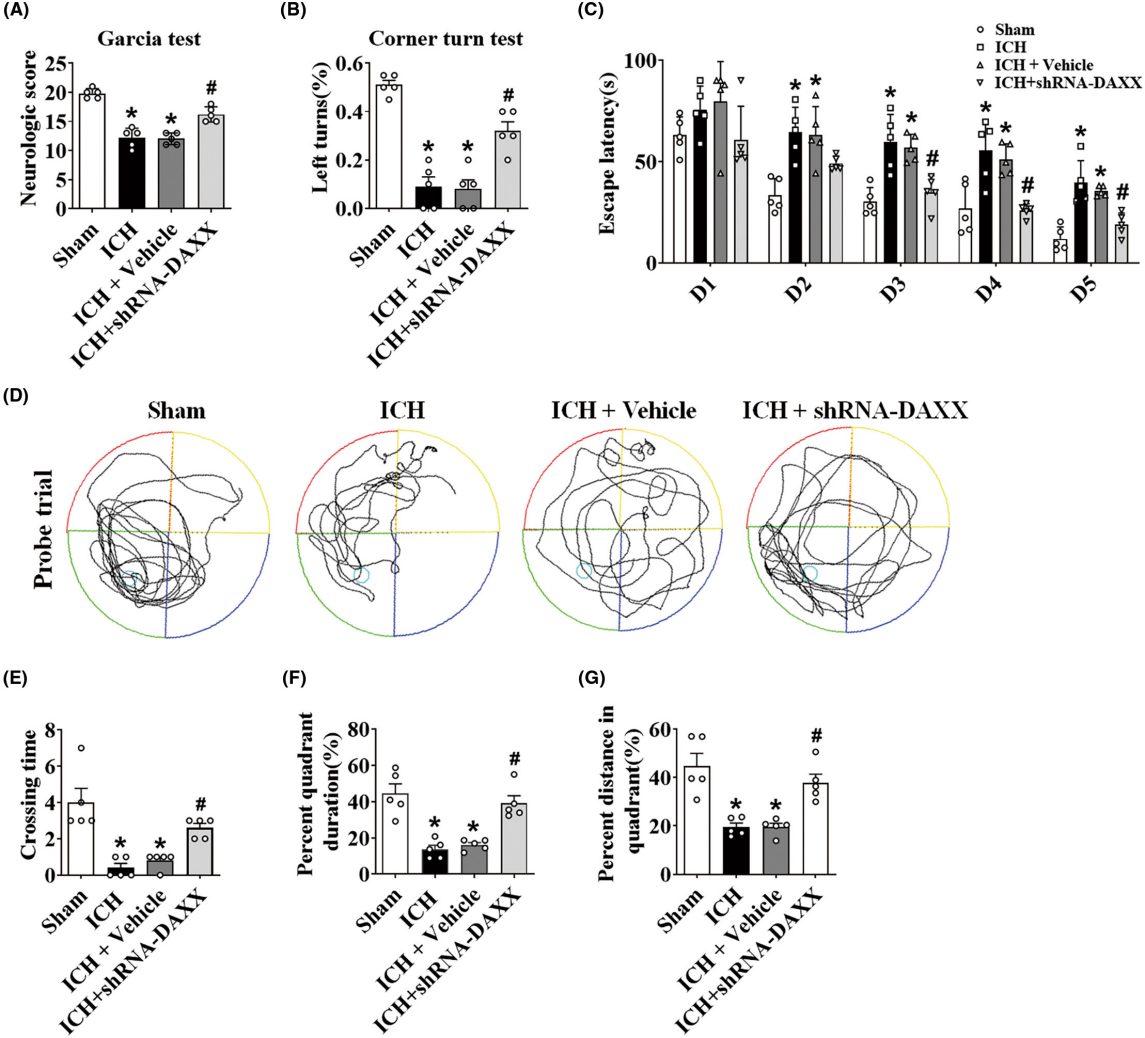
shRNA‐DAXX treatment alleviated the neurological impairments caused by ICH. (A) Garcia test in the sham, ICH, ICH + vehicle, and ICH + shRNA‐DAXX groups (*n* = 5/group). (B) Corner turn test in the sham, ICH, ICH + vehicle, and ICH + shRNA‐DAXX groups (*n* = 5/group). (C) Escape latency in training trials in the sham, ICH, ICH + vehicle, and ICH + shRNA‐DAXX groups (*n* = 5/group). (D) Representative swim traces in probe trials. (E–G) Platform crossing times, percent time in the target quadrant, and percent distance in the target quadrant in probe trials in the sham, ICH, ICH + vehicle, and ICH + shRNA‐DAXX groups (*n* = 5/group). *p < 0.05 compared with sham. ^#^ *p* < 0.05 compared with ICH + vehicle.

Compared to sham mice, ICH and ICH + vehicle animals demonstrated substantial losses in neurological activity. In contrast to those in the ICH + vehicle group, the Garcia test (Figure 6A) and corner turn test (Figure 6B) were significantly improved by shRNA‐DAXX treatment. Neurological performance was examined on Day 28 post‐ICH to test the impact of long‐lasting brain damage. In the training trial, the escape latency of the ICH group was considerably longer than that of the sham group. However, compared to that in the ICH + vehicle group, shRNA‐DAXX treatment dramatically reduced the escape latency on days 3–5 of training (Figure 6C). During the probing trial, the ICH group had shorter platform crossing times, percent time, and percent distance than the sham group. In contrast to the ICH + vehicle group, shRNA‐DAXX treatment significantly increased the time spent in the platform crossing times (Figure 6E), percent time (Figure 6F), and percent distance (Figure 6G).

## 4 DISCUSSION

We were able to compile the emergent molecular landscape in the acute phase by profiling peripheral blood samples of ICH patients. ICH alters the transcriptomic profile. The alterations persisted throughout the formation of brain injury and subsequent expansion. DEGs were significantly associated with necroptosis signaling by pathway and GO analysis. This landscape also showed that DAXX appeared to be a prominent regulator of brain injury. In patients with ICH serum and mouse ICH brain tissues, DAXX was elevated. It was increased by 12 h in ICH mice. Neurological deficits in ICH mice suggested that DAXX aggravated nerve damage. DAXX has been described in brain ischemia/reperfusion injury,^14^ Alzheimer's disease,^19^ myocardial reperfusion injury,^20^ pancreatic neuroendocrine tumors,^21^ and colorectal cancer.^22^ However, it is difficult to generalize other findings to the brain. This work provides important insights into the molecular genesis of brain injury following ICH.

The potential therapeutic value of necroptosis has been recognized in the treatment of a range of illnesses, such as cardiovascular disease,^23^ infectious disease,^24^ renal disease,^25^ and neurodegenerative diseases.^26^ RIPK3 belongs to the RIP kinase family and is an essential inflammatory adapter. It induces inflammation and immunoreactions through necroptosis and other functions.^27^ RIPK3 participates in the nervous system.^28^ The ZBP1/RIPK3 axis limits neuronal viral infection.^29^ RIPK3 promotes cell necroptosis in a spinal cord injury model.^30^ However, no researches have been reported on the activation of RIPK3/DAXX‐mediated neuronal necroptosis signaling during ICH development. We discovered that RIPK3 and DAXX were triggered in the brains of ICH mice. These factors were localized in the majority of the neurons. GSK872, a selective RIPK3 kinase inhibitor, interacts with the RIPK3 kinase domain and suppresses the activity of the enzyme via minimal cross‐reactions.^31^ GSK872 prevented necroptosis and reduced the level of DAXX. Overall, these data indicated that RIPK3 was increased in the acute phase as a reaction to the ICH‐induced necroptosis cascade.

DAXX is involved in a variety of cellular processes, both ATP independent and ATP dependent. The biological effect of DAXX on ICH is still not fully understood. DAXX was first recognized as an adapter protein connected to the Fas (also known as CD95 or Apo‐1) apoptotic receptor's intracellular death domain.^32^ ATRX and DAXX are members of the same ATP‐dependent chromatin remodeling complex and are found in the PML‐nuclear body (PML‐NB).^33^ Partial ATP depletion causes the fast activation of DAXX, Fas, and JNK phosphorylation and apoptosis in animals with ischemia‐induced acute kidney damage.^34^ DAXX, a polyD/E protein, has recently been discovered to be an efficient molecular chaperone that inhibits aggregation, dissolves previously present aggregates, and unfolds misfolded kinds of model substances and proteins linked to dementia. These activities of DAXX depend on the polyD/E region rather than ATP.^19^ In this research, we found that shRNA‐DAXX reduced MLKL and p‐MLKL levels in the brain following ICH in mice. Following ICH, treatment with shRNA‐DAXX enhanced neurological performance and decreased cerebral edema. The striatal area is immediately damaged by ICH, resulting in decreased sensorimotor function.^35^ Following ICH, the hippocampal CA1 area suffers secondary cerebral ischemia.^36^ Human and mammalian learning and memory activities are linked to neurons in the hippocampal CA1 region.^37^ shRNA‐DAXX therapy attenuated neuronal death, which was related to spatial learning deficiencies, as detected by the MWM test. These findings suggest that DAXX is an upstream signal of MLKL revitalization. It regulated MLKL‐dependent neuronal necroptosis after ICH.

Our previous study showed that RIPK3 induced AIF expression and its nuclear translocation after intracerebral hemorrhage injury.^8^ During I/R injury, AIF binds to RIPK3 in the cytoplasm, which is subsequently transported to the nucleus.^38^ In our experiment, we utilized nuclear fractionation to show that the nuclear levels of RIPK3, AIF, and DAXX increased following ICH. Double immunofluorescence analysis showed that DAXX localized in the nucleus. RIPK3 and AIF integrated with nuclear DAXX, respectively, after ICH. During ICH, we applied immunoprecipitation to determine how the DAXX‐RIPK3 and DAXX‐AIF complexes developed. In the ICH group, these complex's extremely enriched IP bands were observed. In response to GSK872 treatment, these bands were blank, indicating that RIPK3 was not functional without nuclear colocalization. This finding indicated that RIPK3 and AIF were bound up with DAXX, respectively, in the nucleus following ICH.

This study has several limitations. The expression profile of DAXX in human perihematomal tissues is still unclear at the stage of ICH. In addition, DAXX expression from the periphery was recruited into the injured brain after ICH. Whether DAXX directly regulates brain injury following ICH requires further investigation.

## 5 CONCLUSION

RIPK3 activation promoted DAXX‐dependent neuronal necroptosis and neurological deficits after ICH. The binding of RIPK3 and AIF to nuclear DAXX is a primary step in necroptosis. Thus, DAXX may be a therapeutic target to increase hematoma absorption and provide insight into ICH treatment.

## AUTHOR CONTRIBUTIONS

Yang Xu conceived the concept of the project; Yang Xu, Qingqing Bai, and Dijing Yu designed the studies; and Qingqing Bai, Shuoyang Wang, Dongmei Rao, Zhiming Zhou, Jianfei Wang, Qi Wang, and Yu Qin performed the experiments. Qingqing Bai, Shuoyang Wang, and Dongmei Rao performed the initial data analysis. Zhaohu Chu and Shoucai Zhao participated in drawing and typesetting. Qingqing Bai drafted the manuscript. Yang Xu and Dijing Yu performed the final analysis of the data and wrote the manuscript. All authors approved the final manuscript.

## FUNDING INFORMATION

This investigation was funded by grants from National Natural Science Foundation of China (82,171,329; 81,701,161); Chinese Stroke Society Metabolomics Fund for Cerebrovascular Disease; Education department of Ahhui Province, China (KJ2021ZD0096); Wannan Medical College, Anhui, China (YR201802, KGF2019G02, WK2022F07, WK2022F23, WK2022F16); Ahhui Excellent Young Talents Suppoot Program in University; Anhui Higher Education Institution's Key Laboratory of Non‐coding RNA Transformation Research's Open Project (RNA202201); Clinical Medical Research Translational Project (202204295107020017). We thank X‐way group for designing this research.

## CONFLICT OF INTEREST STATEMENT

The authors declare no competing interests.

## Supporting information


Data S1.

## Data Availability

The data supporting the findings of the present study are available from the corresponding authors upon reasonable request.
