# Supplementary material for: RIPK3 activation promotes DAXX‐dependent neuronal necroptosis after intracerebral hemorrhage in mice
Source: CNS Neurosci Ther. 2023 Aug 8;30(1):e14397. doi: 10.1111/cns.14397 (PMC10805394; doi:10.1111/cns.14397)
Supplement: Supplementary file 1 — Data S1. [file CNS-30-e14397-s001.docx]

**RIPK3 activation promotes DAXX-dependent neuronal necroptosis after intracerebral hemorrhage in mice**

**Supplementary Information**

**Supplementary Methods and Materials**

**Inclusion and exclusion criteria**

All patients were from the cohort of Chinese cerebral hemorrhage: mechanism and intervention study, and met the following inclusion criteria: (1) Over 18 years of age; (2) Patients were diagnosed with ICH according to the guidelines; (3) Within 7 days of onset and will rule out when meeting one of the exclusion criteria: (1) Subarachnoid hemorrhage; (2) Hemorrhagic transformation after cerebral infarction; (3) Bleeding after thrombolysis; (4) Traumatic intracerebral hemorrhage; (5) Epidural hematoma and subdural hematoma.

Healthy control subjects were recruited from healthy examination subjects. Exclusion

criteria include a history of head trauma, major diseases, and drug abuse.

**Cell culture**

HT22 cells were purchased from EK-Bioscience Biotechnology (CC-Y2137, EK-Bioscience, China). Cells were transferred to a DMEM medium containing 10% fetal bovine serum and 1% penicillin/ streptomycin at 37°C with 5% CO2 ^[1]^.

**Experimental protocol**

**Experiment 1**

12 mice were randomly divided into 4 groups (n = 3/group): sham, 12 h, 24 h, and 48 h after ICH. Western blotting was performed to evaluate the time course of RIPK3, AIF, DAXX, MLKL, and p-MLKL expression. Additional mice included in the 24 h post-ICH group were used for double immunofluorescence to assess the cellular localization of RIPK3, AIF, and DAXX in the brain. An additional 6 mice were randomly divided into 2 groups (n = 3/group): sham and 24 h post-ICH groups. Double immunofluorescence to assess the cellular localization of RIPK3, AIF, and DAXX in the brain.

**Experiment 2**

To elucidate the effect of GSK872 treatment on the level of DAXX during ICH, 12 mice were randomly divided into 4 groups (n = 3/group): sham, ICH, ICH + Vehicle, and ICH + GSK872. The RIPK3 inhibitor GSK872 was administered intracerebroventricularly 1 h before ICH. Western blot analysis was examined at 24 h after ICH.

**Experiment 3**

To further explore the effect of shRNA-DAXX on the level of MLKL, brain edema, and neurobehavior following ICH, 72 mice were randomly divided into 4 groups (n = 18/group): sham, ICH, ICH + Vehicle, and ICH + shRNA-DAXX. The Vehicle or DAXX inhibitor shRNA-DAXX was injected intracerebroventricularly at 2 weeks before ICH. Western blot analysis was examined at 24 h after ICH. At 24 h post-ICH, the brain edema and neurobehavior tests were conducted. On Day 28 post-ICH, the Morris water maze test was performed.

**Experiment 4**

To determine the association between DAXX, RIPK3, and AIF, 18 mice were randomly divided into 3 groups (n = 6/group): sham, ICH, and ICH + GSK872. In each group, mice were selected for immunofluorescence staining and immunoprecipitation.

Random sequences were generated by computer. The allocation sequence was concealed from the investigators in sequentially numbered, opaque, and sealed envelopes. Sham and ICH mice that immediately survived the surgery were included.

The exclusion criteria included the absence of hemorrhage in ICH mice and death prior

to/during surgical procedures. All experimental group/treatment information was blinded to the researchers who performed the neurobehavioral tests and data analysis.

**ICH brain tissue harvesting**

Animals were transcardially perfused with ice-cold phosphate-buffered saline (PBS) after anesthesia. As described previously ^[2]^, the whole brain or tissue around the hematoma was separated and stored in a freezer at -80℃ for subsequent protein analysis and RNA isolation later.

**Hematoxylin and eosin staining**

Mouse brain tissues were rapidly removed at 24 h after ICH and fixed with 4% paraformaldehyde for 24 h. After paraffin embedding, mouse brain tissues were prepared on 4 μm coronal sections and stained with hematoxylin and eosin. We observed the number of surviving hippocampal CA1 neuronal cells under a light microscope (Olympus IX81, Tokyo, Japan).

**
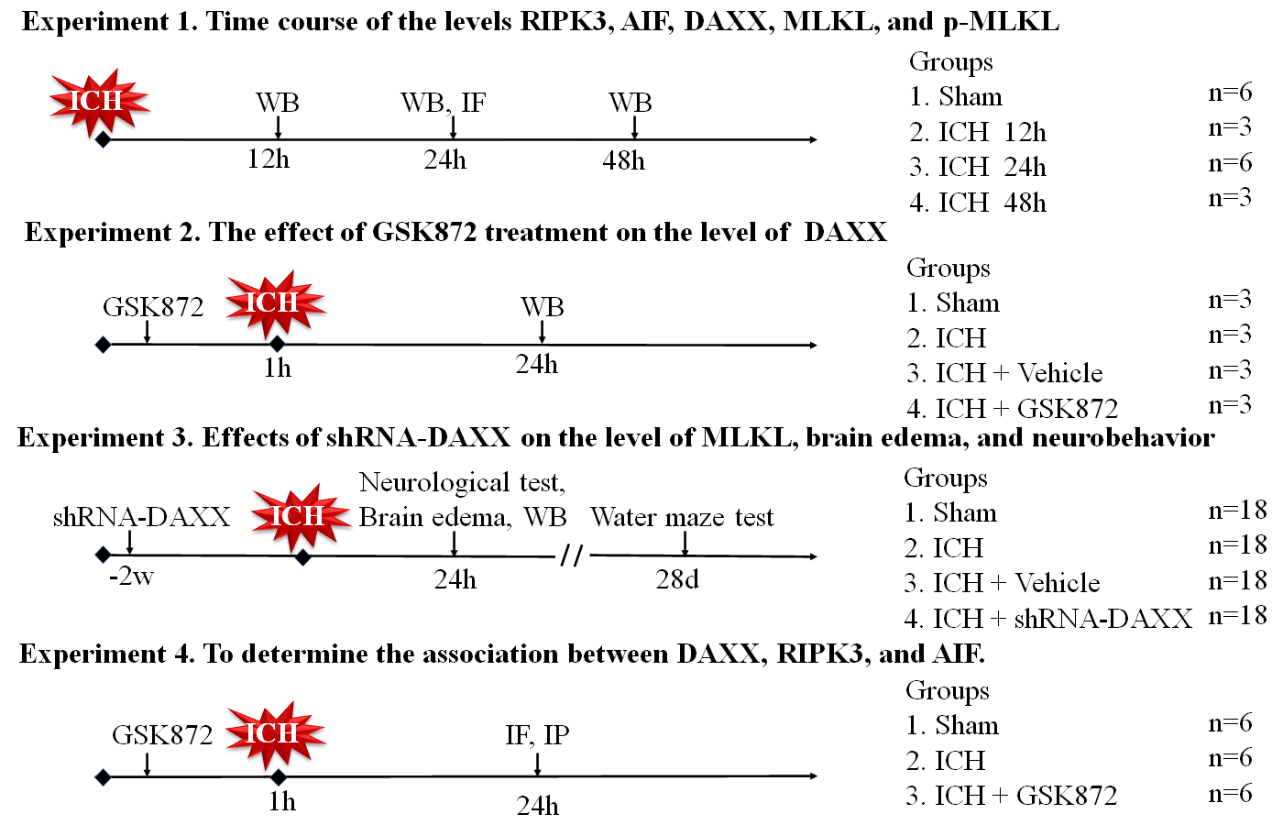
**

**Supplemental Figure S1.** Study design.

Representative figure showing the experimental design and number of animals for each group. ICH, intracerebral hemorrhage; WB, western blot; IF, immunofluorescence; IP, immunoprecipitation.





**Supplemental Figure S2.** Time course of serum DAXX and RIPK3 expression in ICH patients.

(A) Quantitative real‑time PCR of DAXX mRNA expression in the serum of patients with ICH (n = 5) compared with that in healthy control participants (n = 5). GAPDH was used as an internal control. (B) Enzyme-linked immunosorbent assay results showed the level of DAXX in the serum after ICH patients (n = 5) and control participants (n = 5). (C) Quantitative real‑time PCR of RIPK3 mRNA expression in the serum of patients with ICH (n = 5) compared with that in healthy control participants (n = 5). GAPDH was used as an internal control. (D) Enzyme-linked immunosorbent assay results showed the level of RIPK3 in the serum after ICH patients (n = 5) and control participants (n = 5). **P* < 0.05 compared with healthy control participants.

**
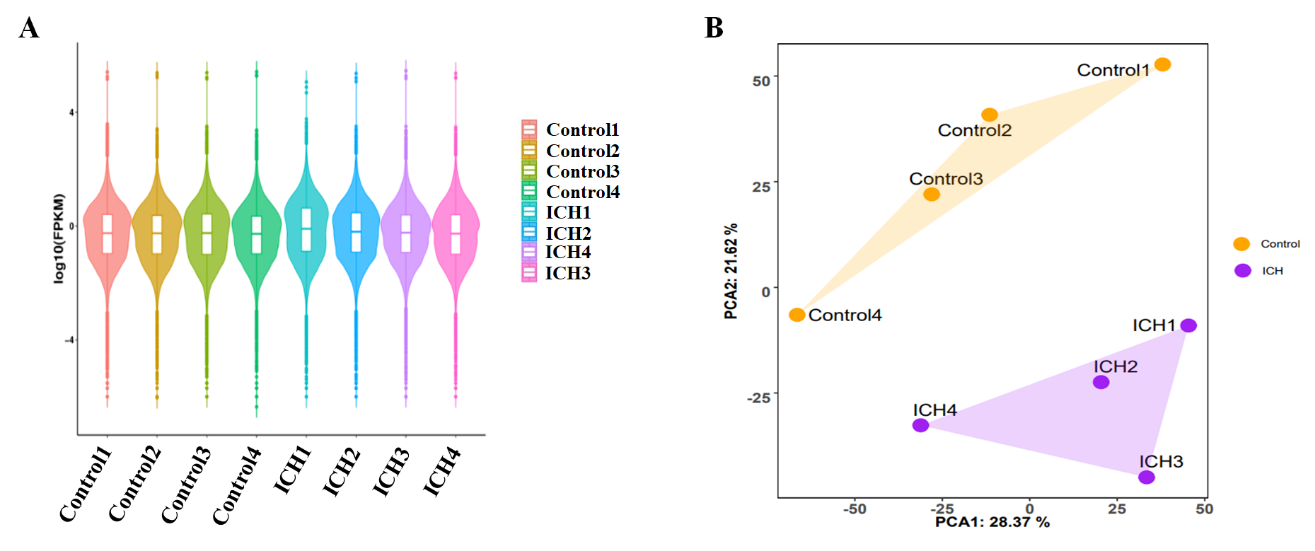
**

**Supplemental Figure S3.** Quantification and quality control of RNA sequencing data.

(A) The box plot of fragments per kilobase of transcript sequence per millions mapped reads distribution per sample (n =4/group). (B) Principal component analysis based on variance stabilizing transformation of molecule counts: each symbol corresponds to one patient. Localization is highlighted by a color (purple = ICH, orange=control, n = 4/group).


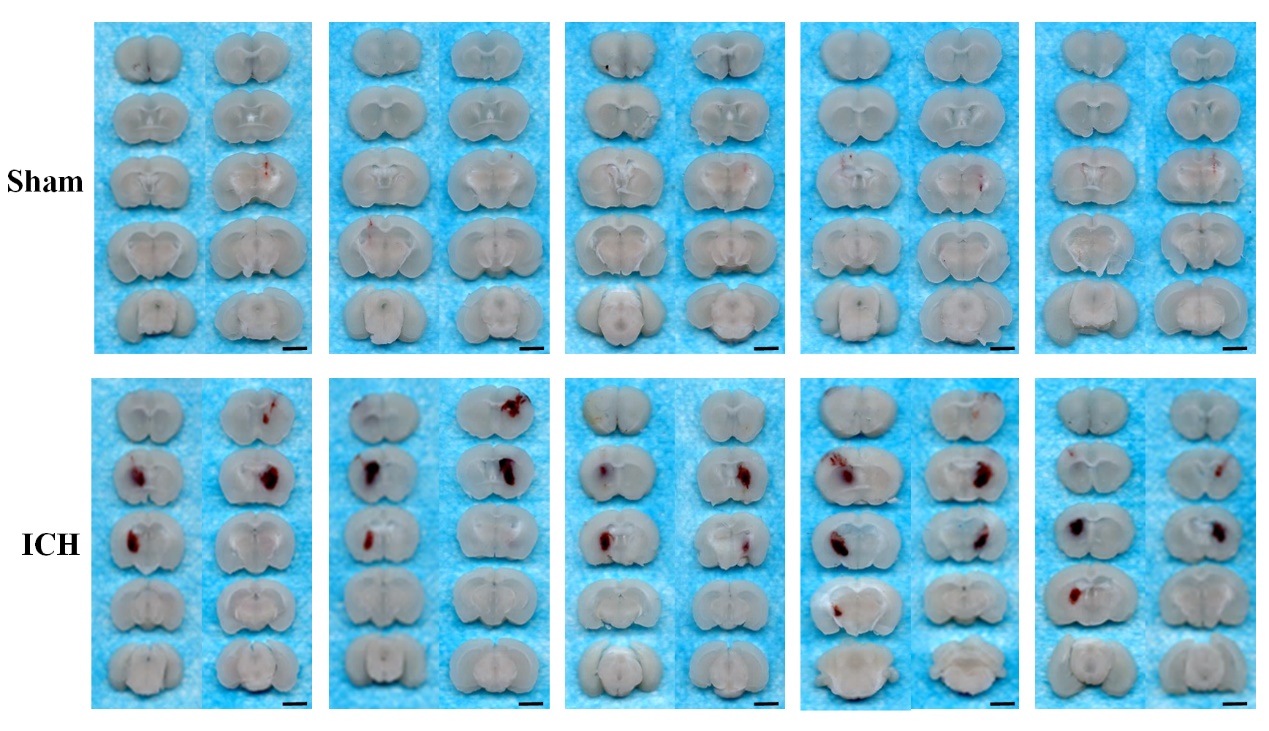


**Supplemental Figure S4.** Images represented sham and ICH coronal brain sections at 24 h after ICH. Scale bar = 5 mm, (n = 5/group).


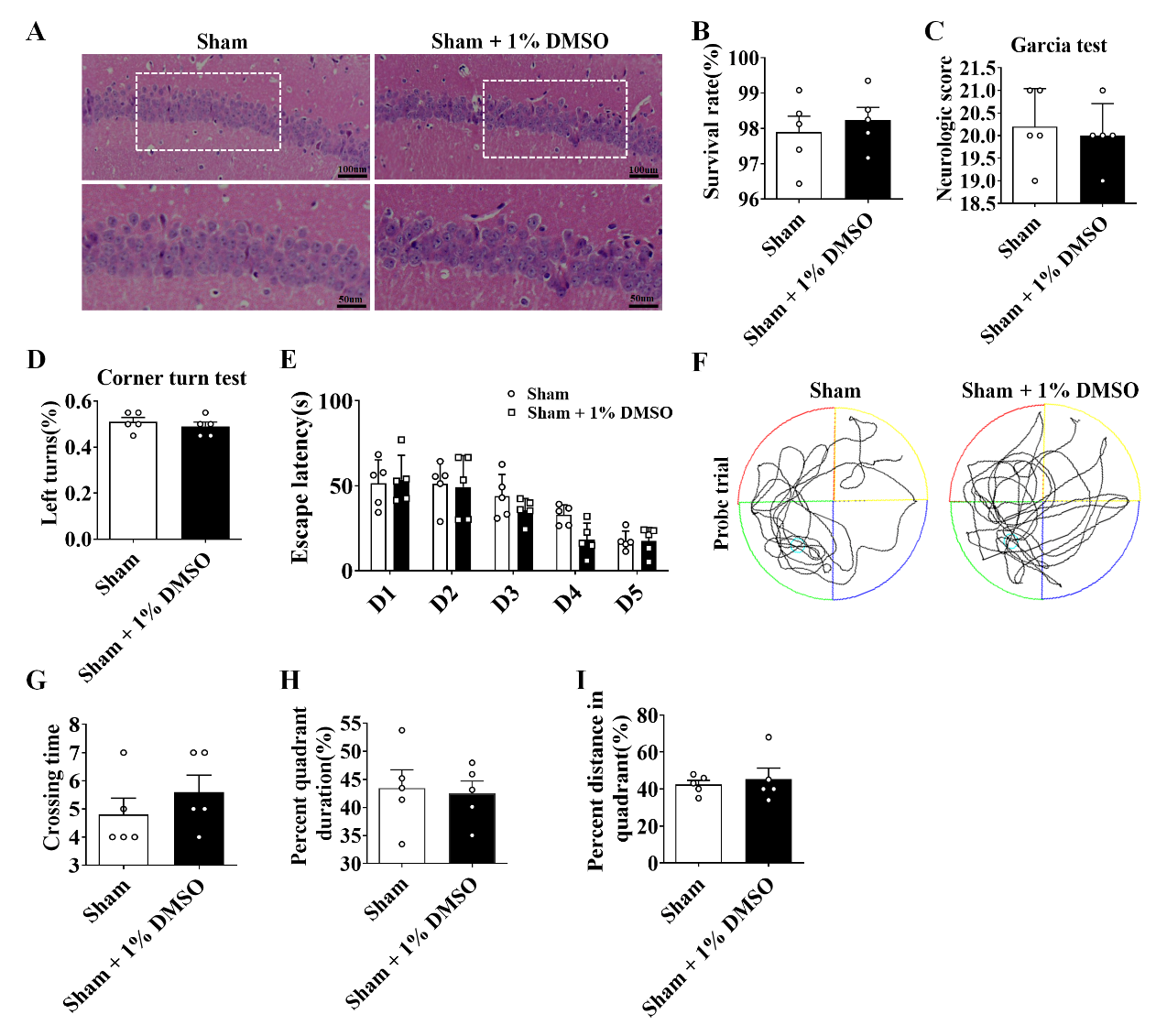


**Supplemental Figure S5.** 1% DMSO had no toxic effect on the brain.

(A). Representative hematoxylin and eosin staining of hippocampal CA1 neurons from the sham group and the sham + 1% DMSO group. (B) Neuronal survival rates in the hippocampal CA1 region in the sham group and the sham + 1% DMSO group. (n=5/group). (C) The Garcia test in the sham group and the sham + 1% DMSO group (n = 5/group). (D) The corner turn test in the sham group and the sham + 1% DMSO group (n =5/group). (E) Escape latency in training trials in the sham group and the sham + 1% DMSO group (n = 5/group). (F) Representative swim traces in probe trials. (G-I) Platform crossing times, percent time in the target quadrant, and percent distance in the target quadrant in probe trials in the sham group and the sham + 1% DMSO group (n = 5/group).


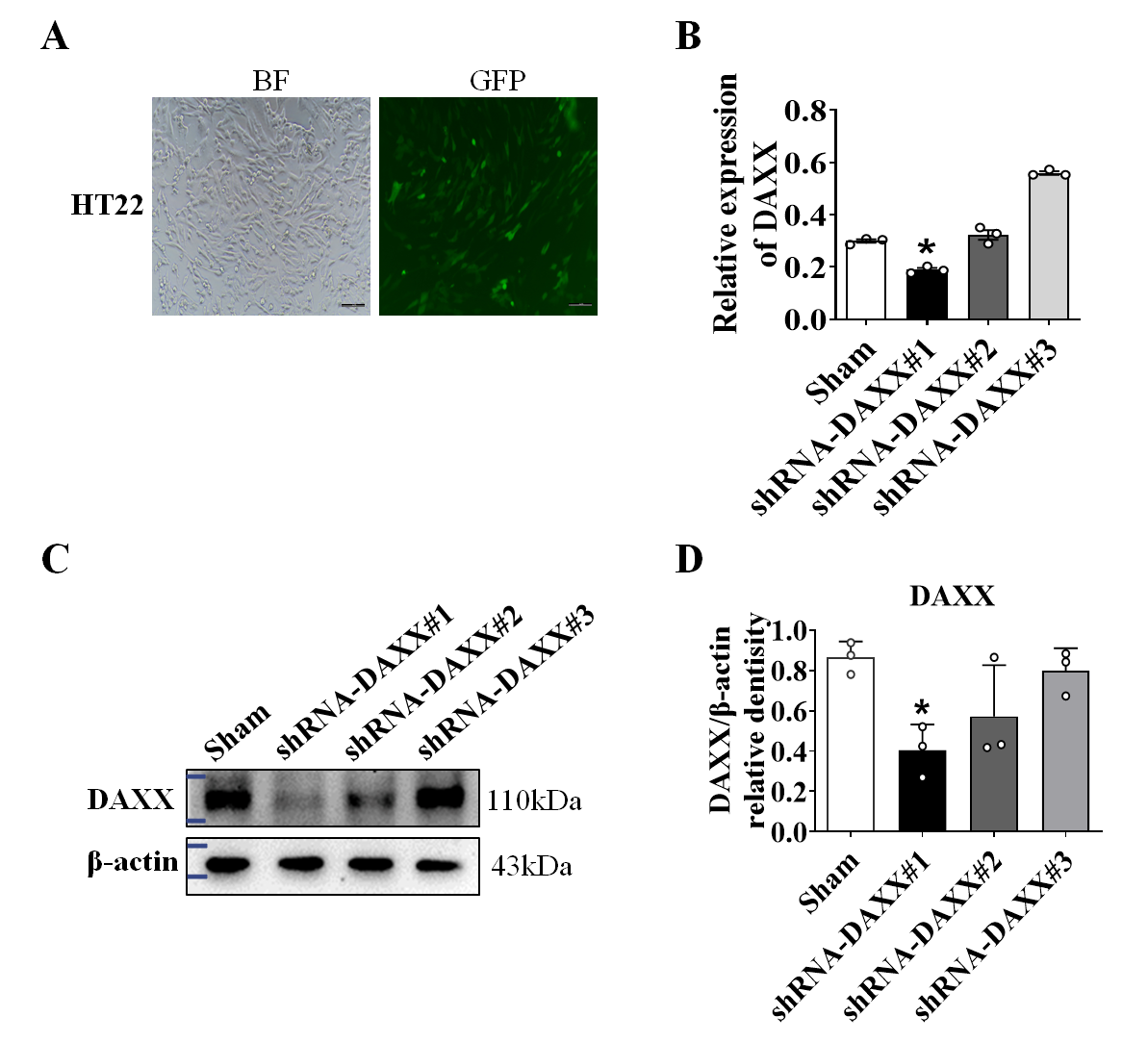


**Supplemental Figure S6.** Three DAXX-specific shRNAs were used to knock down endogenous DAXX expression in HT22 cells.

(A) Bright-field (BF) and fluorescent (GFP) microscopy showing normal HT22 cells transduced with shRNA-Scramble-GFP lentivirus. (B) Quantitative real‑time PCR of DAXX mRNA expression in the sham, shRNA-DAXX#1, shRNA-DAXX#2, and shRNA-DAXX#3 group (n = 3/group). (C) Representative Western blot images. (D) Quantitative analyses of DAXX in the sham, shRNA-DAXX#1, shRNA-DAXX#2, and shRNA-DAXX#3 group (n = 3/group). **P* < 0.05 compared with sham.


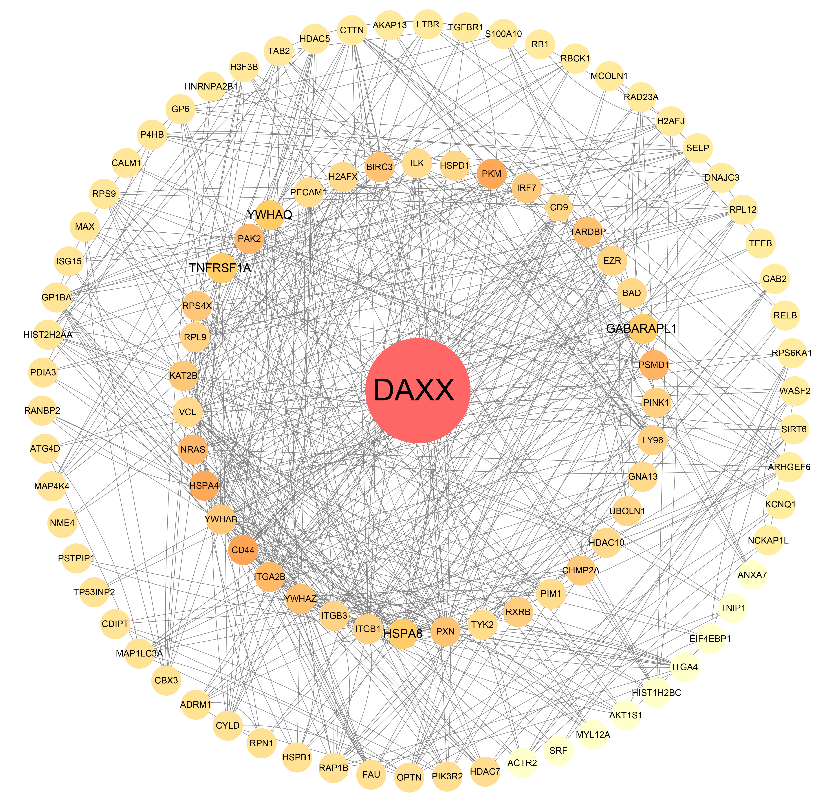


**Supplemental Figure S7.** DAXX was closely interconnected with others in the functional PPI network.


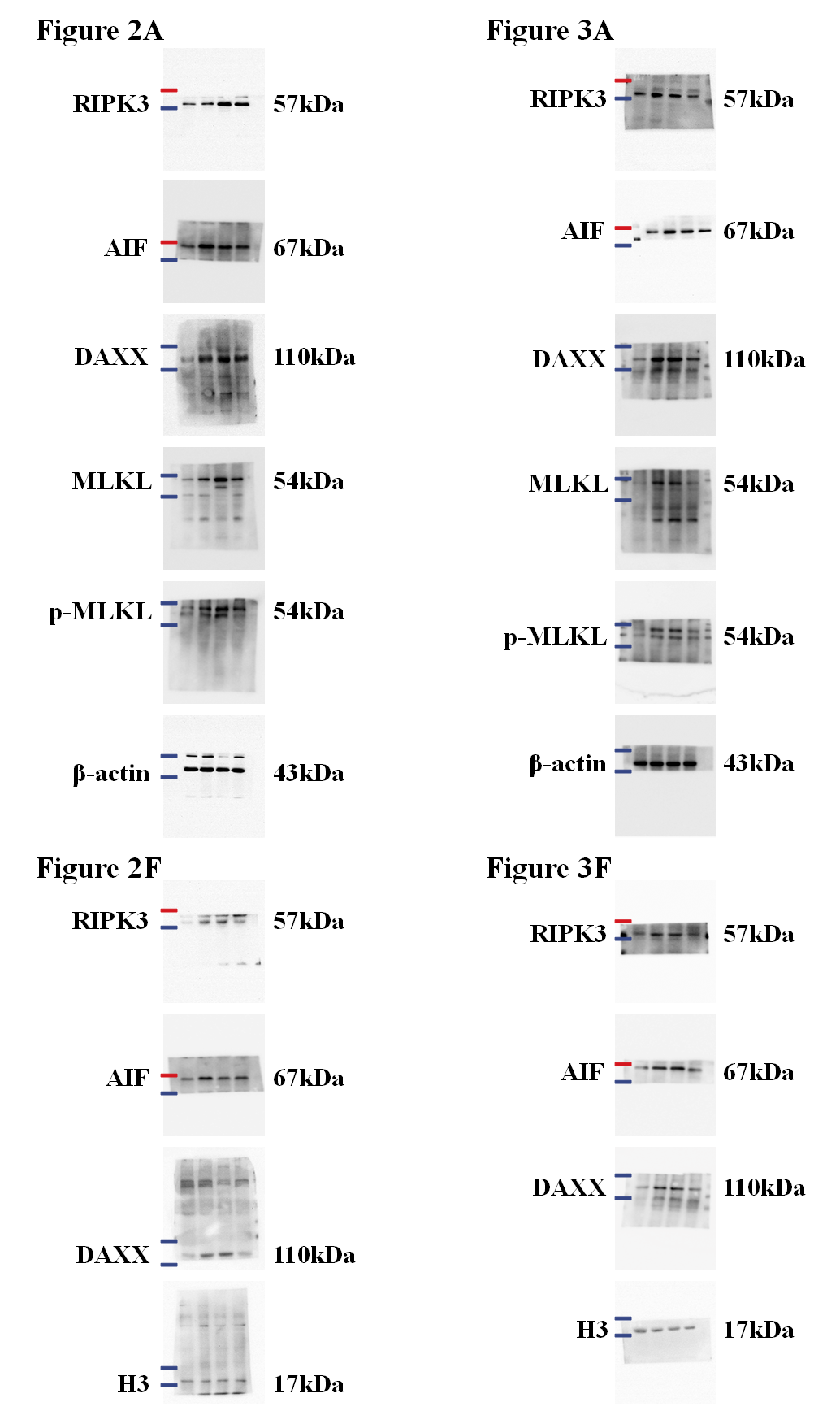


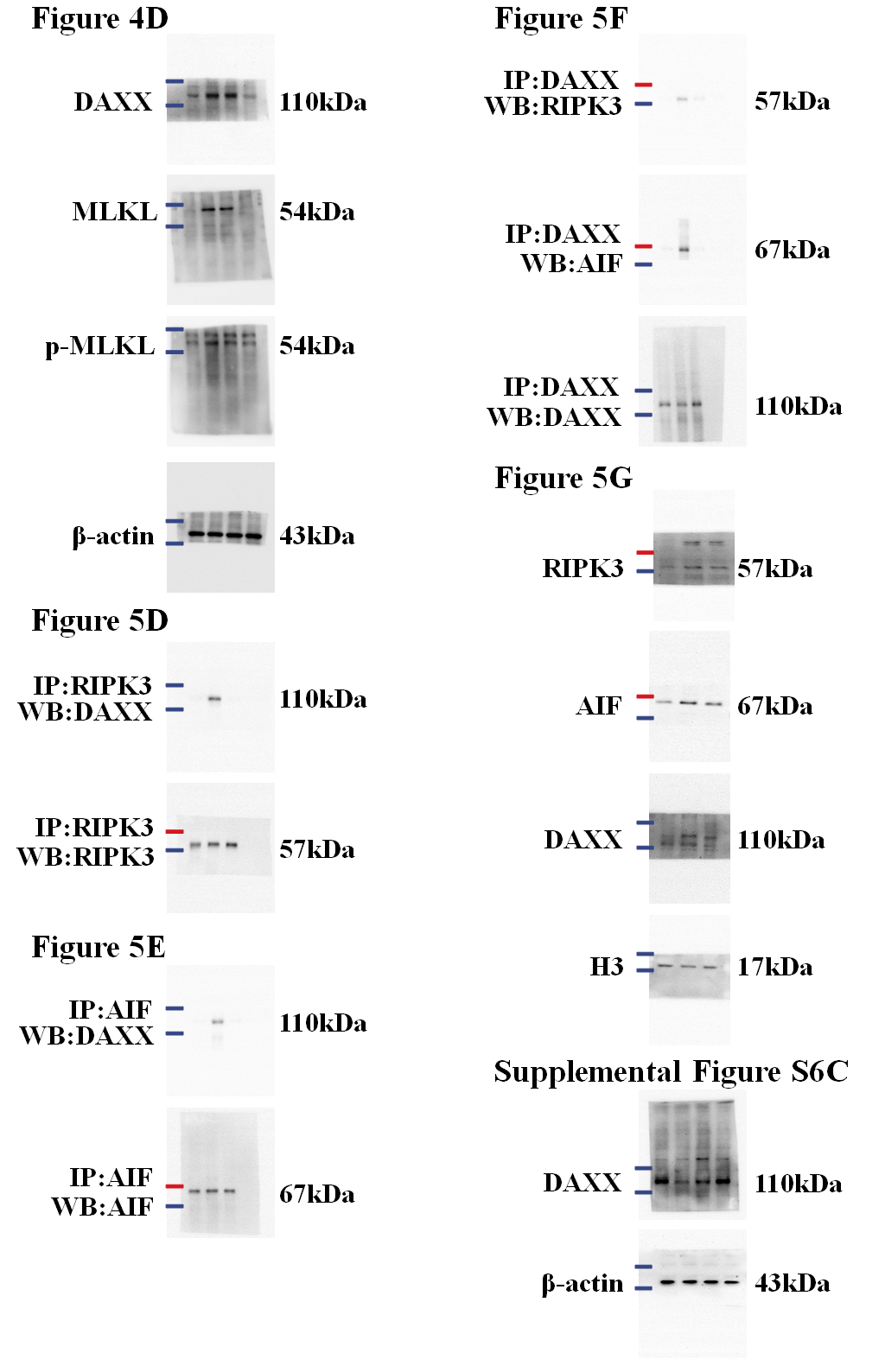


**Supplemental Figure S8**. The original gel/blot images of each figure.

**Supplemental Table S1.** Baseline characteristics of the 61 included patients

| **Characteristic** | **ALL**  N = 61 | **ICH group**  N = 34 | **Control group**  N = 27 | **Statistical**  **test** | ***P*-value** |
| --- | --- | --- | --- | --- | --- |
| **Age, y** (SD) | 62.32(14.02) | 63.48(14.2) | 61.85(9.42) | 0.157 | 0.876 |
| **Sex, male**, n (%) | 37(60.7) | 25 (73.5) | 12(44.4) |  | 0.021 |
| **Medical History,** n (%) |  |  |  |  |  |
| Hypertension | 46(75.4) | 31(91.2) | 15(55.6) |  | 0.001 |
| Diabetes **Mellitus** | 14(23.0) | 13(38.2) | 1(3.7) |  | 0.003 |
| Cardiology | 7(11.5) | 5(14.7) | 2(7.4) |  | 0.448 |
| History of Stroke | 7(11.5) | 5(14.7) | 2(7.4) |  | 0.448 |
| Hyperlipemia | 15(24.6) | 8(23.5) | 7(25.9) |  | 0.829 |

ICH, intracerebral hemorrhage

**References**

1. Nasoni MG, Carloni S, Canonico B, et al. Melatonin reshapes the mitochondrial network and promotes intercellular mitochondrial transfer via tunneling nanotubes after ischemic-like injury in hippocampal HT22 cells. *J Pineal Res*. 2021;71(1):e12747.

2. You M, Long C, Wan Y, et al. Neuron derived fractalkine promotes microglia to absorb hematoma via CD163/HO-1 after intracerebral hemorrhage. *Cell Mol Life Sci*. 2022;79(5):224.
